# Effects of Rearing Density on Developmental Traits of Two Different Biotypes of the Gypsy Moth, *Lymantria Dispar* L., from China and the USA

**DOI:** 10.3390/insects12020175

**Published:** 2021-02-17

**Authors:** Yiming Wang, Robert L. Harrison, Juan Shi

**Affiliations:** 1 Sino-France Joint Laboratory for Invasive Forest Pests in Eurasia, Beijing Forestry University, Beijing 100083, China; iris_wym@163.com; 2 Invasive Insect Biocontrol and Behavior Laboratory, USDA-ARS, Beltsville, MD 20705, USA; Robert.L.Harrison@usda.gov

**Keywords:** quarantine pests, gypsy moth, *Lymantria dispar*, larval density, density-dependent development

## Abstract

**Simple Summary:**

The gypsy moth, *Lymantria dispar*, is a forest pest that is grouped into two biotypes: the European gypsy moth (EGM), found in Europe and North America; and the Asian gypsy moth (AGM), found in China, Russia, Korea, and Japan. Outbreaks of this pest result in high-density populations of larvae that can cause enormous damage to trees and forests. Studies have identified an influence of larval population density on gypsy moth development, but it is not known how EGM and AGM populations differ in their response to changing larval densities. We examined the effects of varying larval density on three colonies established from one EGM population and two AGM populations. All three colonies exhibited an optimal degree of larval survival and optimal rates of pupation and adult emergence at an intermediate density of five larvae/rearing container. The duration of larval development was fastest at the same intermediate density for all three colonies. Although differences in larval development time, survival, pupation and emergence were observed among the three colonies under the conditions of our study, our findings indicate that density-dependent effects on the development of different gypsy moth biotypes follow the same trends.

**Abstract:**

The life-history traits of the gypsy moth, *Lymantria dispar* L. (Lepidoptera: Erebidae), have been observed to vary with larval population density, which can increase significantly during an outbreak of this pest. Laboratory studies on density-dependent variation in gypsy moth development have focused on single populations and were limited to comparing solitary larvae with groups of larvae reared at a single density. To evaluate how density-dependent impacts on development vary with different populations and subspecies of *L. dispar*, we compared the effects of rearing larvae of a European gypsy moth (*L. dispar dispar* L.) population from Connecticut, USA; and larvae of two populations of the Asian gypsy moth (*L. dispar asiatica* Vnukovskij) from Guizhou and Hebei provinces in China. Larvae were reared on an artificial diet at densities of one, three, five, seven, and nine larvae per 115 mL container, and the duration of larval development, percentage of surviving larvae, and the rates of pupation and emergence were measured at each density. A two-tailed response to density variation with values falling away on both sides from a peak or climbing from a base was observed for all three populations tested, with the most rapid larval development and the highest values of survival, pupation, and emergence observed at a density of five larvae/container. Although differences in larval development time, survival, pupation and emergence were observed among the different populations under the conditions of our study, our findings indicate that density-dependent effects on the development of different gypsy moth subspecies and populations follow the same trends.

## 1. Introduction

A quarantine pest is defined by the Food and Agriculture Organization of the United Nations (FAO) [1] as a “pest of potential economic importance to the area endangered thereby and not yet present there, or present, but not widely distributed and being officially controlled.” Quarantine insect pests are a major threat to the ecology and environment that can cause huge economic losses [2]. For example, three common quarantine insect pests in forests—the hemlock woolly adelgid, the European gypsy moth, and the emerald ash borer—have been reported to cause damage to the forestry ecosystem worth approximately $2.1 billion dollars per year in the United States [3]. As one of the most destructive defoliating forest pests, the gypsy moth, *Lymantria dispar* (L.) (Lepidoptera: Erebidae), is a quarantine pest species with two major biotypes distributed throughout the world. One of them is the European gypsy moth, *Lymantria dispar dispar* (L.), which can be found in Europe and North America. The other one is the Asian gypsy moth, including subspecies *Lymantria dispar asiatica* (Vnukovskij) in mainland East Asia and *Lymantria dispar japonica* (Motschulsky) in Japan. Compared with the European gypsy moth, the Asian gypsy moth has been considered to be a more dangerous pest species due to its stronger female flight ability [4] and the potential to subsist on a different, potentially wider range of hosts [5]. In addition, these two biotypes have distinct developmental traits such as survival and developmental duration at different life stages [5]. Although the Asian gypsy moth is not yet established in North America and Europe, considering its global invasive potential, it has been listed as one of the most important quarantine insect pests by the North American Plant Conservation Organization (NAPPO) [6,7].

Gypsy moth populations oscillate between a low-density (“innocuous”) phase and a high-density phase observed during outbreaks [8,9,10,11]. Changes in larval density are associated with a variety of positive and negative effects on the life-history traits of insects [12,13]. While the response to higher larval rearing densities varies from one species to the next, higher density is generally associated with slower larval developmental times, lower larval and adult survival, lower body mass, reduced adult fecundity, and other morphological and physiological changes. These observations can, in part, be attributed to the competition for limited food resources among larvae. This mechanism is suggested by a study on the butterfly, *Bicyclus anynana*, which exhibited faster (rather than slower) larval development at a high larval rearing density when food quantity was not limiting [14].

In laboratory studies on the gypsy moth, higher larval densities have been associated with either a shorter or longer larval development time, lower larval mass, higher larval digestive enzyme activity, higher larval hemocyte concentration, lower larval dopamine concentration, higher larval mortality, and lower pupal weights [15,16,17,18]. These studies examined single populations of either *L. dispar dispar* or *L. dispar asiatica*. The effects of larval density have not been consistent among different species of Lepidoptera [13], which raises the question of whether changes in larval density have the same effect on different subspecies and biotypes of Lepidopteran species such as *L. dispar*. In addition, past studies on density-dependent variation in gypsy moth development have compared larvae reared in isolation with larvae reared at a single defined “crowded” density. In this study, we set out to see if density-dependent effects on life-history traits differ among different biotypes and populations of *L. dispar* and to determine how these effects vary with a range of different rearing densities.

## 2. Materials and Methods

### 2.1. Gypsy Moth Populations

Colonies established from three populations of gypsy moths were used in this study, including two populations of Asian gypsy moths collected from Guizhou Province in southern China (106°56’ E, 27°42′ N) and Hebei Province in northern China (115°52′ E, 41°00′ N), respectively, and one population of the European gypsy moth collected in Connecticut, the United States (72°41′ W, 41°37′ N). The two populations of Asian gypsy moths were collected as fresh eggs from the field in 2017 and reared in the laboratory. The European gypsy moth was obtained in 2017 and shipped as fresh egg samples from the USDA-APHIS-PPQ (US Department of Agriculture-Animal and Plant Health Inspection Service-Plant Protection and Quarantine). Once received, European gypsy moths were reared in the quarantine facility at Beijing Forestry University. These three populations were used in previous studies documenting their genetic diversity [19,20,21].

### 2.2. Diet and Rearing Conditions

All populations were reared in the laboratory on an artificial diet prepared as described [22]. All experiments were conducted in incubators with controlled parameters (temperature: 25 ± 0.5 °C; photoperiod: 14 h light: 10 h dark, and relative humidity: 45 ± 1%).

### 2.3. Rearing Gypsy Moth at Different Densities

To evaluate the density-dependent development of the three gypsy moth populations, neonate larvae were placed in jelly cups (115 mL) with 1.5 cm (average 50 g per cup) of artificial diet at five different densities, including one, three, five, seven, and nine larvae per cup. The jelly cups consisted of food-grade polypropylene cups, which were perforated with a thin needle heated with an alcohol lamp. After perforation, the sides of the cup were lined with a fine mesh to prevent egress of early instar larvae. The cups were inverted during incubation to separate the diet from larval feces for hygienic purposes. To further ensure sufficient food quantity and quality, all larvae were transferred daily to cups with a freshly made diet. For each larval density of each population, three replicates were established, and four variables were recorded, including (1) the duration of larval development, (2) survival rate of larvae, (3) percentage of pupation among surviving larvae, and (4) percentage of emergence. All replicates were examined, and data for the above variables were recorded daily.

### 2.4. Statistical Analysis

To assess statistical differences in developmental traits among the three gypsy moth populations, a two-way factorial analysis of variance (ANOVA) was used, with the population and density being the two factors. The Shapiro–Wilk test was used to confirm the normality of the data (*p* > 0.05). For all significant effects detected, post hoc comparison was conducted using Tukey’s HSD method at α = 0.05. All analyses were conducted in GraphPad Prism 8.0.

## 3. Results

In this study, we compared four developmental traits at five different larval densities for three gypsy moths from distinct geographical regions. In all factorial ANOVA analyses of the data, both the population and density had significant effects on larval development time (population: F (2,30) = 15.0, *p* < 0.0001; density: F (4,30) = 20.73, *p* < 0.0001), larval survival (population: F (2,30) = 53.62, *p* < 0.0001; density: F (4,30) = 109.1, *p* < 0.0001), pupation (population: F (2,30) = 24.46, *p* < 0.0001; density: F (4,30) = 66.51, *p* < 0.0001), and adult emergence (population: F (2,30) = 3.979, *p* < 0.0001; density: F (4,30) = 88.88, *p* < 0.0001), while their interactions had no effects with all *p* -values > 0.05 (Table 1).

### 3.1. Impact on Larval Development Time and Survival

**Figure 1:**
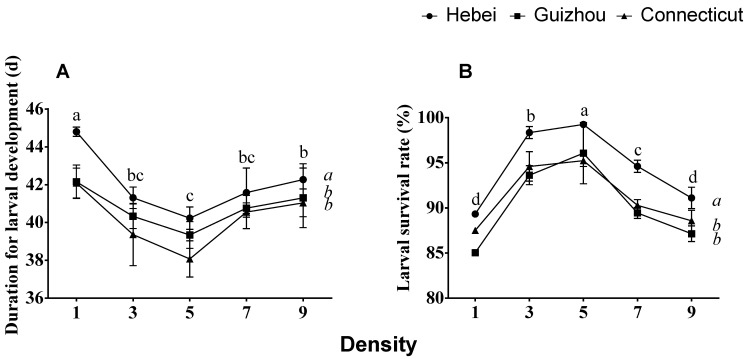
Effects of larval density and geographical population on (**A**) the duration of larval development, in days and (**B**) larval survival (%) of gypsy moths collected from Hebei and Guizhou, China, and Connecticut, USA. The means ± SE for these traits are plotted against larval density. Two-way ANOVA was used, followed by Tukey’s method for the post hoc comparisons at α = 0.05. The populations (italicized letters) and larval densities (letters in normal type) designated with different letters are significantly different.

The duration of larval development and the rate of larval survival exhibited the same response to density for all three populations (Figure 1). Larval development time at a density of five larvae/cup was significantly lower than the development times at one and nine larvae/cup (Figure 1A). The survival rate of larvae was optimal at a density of 5 larvae/cup, with approximately 95–100% of larvae surviving to pupation at this density (Figure 1B). Larval survival was slightly but significantly lower at other densities, with approximately 85–92% of larvae surviving at densities of 1 and 9 larvae/cup. Larvae of the Hebei population exhibited a significantly slower degree of larval development than the Guizhou and Connecticut populations and also fared best with the highest populations of surviving larvae at each density.

### 3.2. Impact on Pupation and Emergence

**Figure 2:**
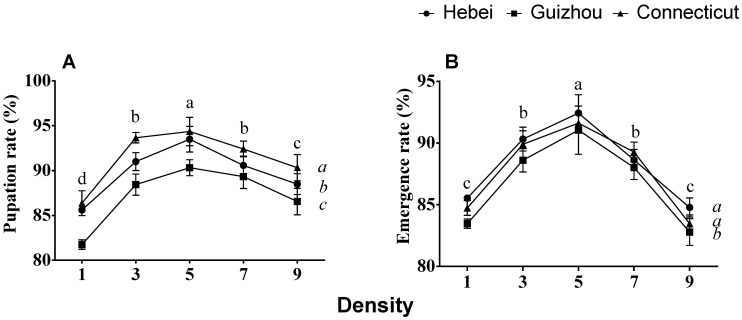
Effects of larval density and geographical population on (**A**) pupation rate (%) and (**B**) emergence rate (%) of gypsy moths collected from Hebei and Guizhou, China, and Connecticut, USA. The means ± SE for these traits are plotted against larval density. Two-way ANOVA was used, followed by Tukey’s method for the post hoc comparisons at α = 0.05. The populations (italicized letters) and larval densities (letters in normal type) designated with different letters are significantly different.

The percentages of pupation and emergence among surviving larvae were also optimal at a density of five larvae/cup, with significantly lower percentages at the other densities (Figure 2). Pupation and emergence percentages were lowest at one and nine larvae/cup. Significant differences in pupation rate were also observed among the three populations, with the Connecticut larvae exhibiting the highest rate of pupation (Figure 2A). The Guizhou larvae exhibited a slightly but significantly lower degree of emergence compared to the larvae of the Hebei and Connecticut populations, with emergence occurring at percentages below 85% at the 1- and 9-larvae/cup densities (Figure 2B).

## 4. Discussion

There is some uncertainty in determining with precision what a “crowded” population would be under laboratory conditions. Leonard [15] identified a larval density as “crowded” when it caused observable differences in gypsy moth development in preliminary experiments. Correlating estimated gypsy moth larval densities (reported, for example, as larvae/ha by Campbell [23]) with larval density in a rearing cup is not straightforward. It is likely that the range of densities used in our study captured at least some of the range of densities that occurs in nature. Gypsy moth densities in larvae/ha estimated by Campbell [23] varied by approximately 60–80-fold among 8 different sites sampled in two consecutive years.

All four measured variables exhibited a two-tailed response characterized by an apparent Allee effect, with optimal larval survival, pupation, emergence, and the fastest larval development time observed for all three populations at five larvae/cup compared to other larval densities below and above five larvae/cup. Although other studies with gypsy moth larvae were limited to comparing larvae reared singly with larvae reared in a single grouping, two-tailed responses also have been reported for larvae of the Lepidopterans *Mamestra brassicae* [24], and *Spodoptera exigua* [25] reared in culture at a series of different densities.

Prior studies reported conflicting trends with regard to larval development times, which were either shorter [15,18] or longer [17] among larvae reared under crowded conditions compared to solitary larvae. The results of these studies likely reflect different responses of larvae at different densities equivalent to the densities in the range tested in our experiments.

The reduced larval development time observed when density increased from one larva to five larvae/cup may be an adaptation to the potential exhaustion of food resources at higher densities, as has been suggested by Bauerfeind and Fischer [14]. Explaining the increased larval development time when larval densities are increased from five to nine larvae/cup is more difficult. Larvae were transferred to fresh cups of diet every day to ameliorate or reduce any possible contribution of reduced food quality or quantity to developmental traits. Excessive physical contact among larvae occurring at higher densities has been speculated to interfere with larval feeding, which would be expected to reduce the rate of larval development [23,24]. However, in an experiment in which crowding and increased larval contact were simulated by adding freeze-dried gypsy moth larvae to rearing cups, there was little effect observed on the duration of larval development [17].

The effects of larval density on susceptibility to pathogens [26] may have also contributed to the trends observed in our data. Reilly and Hajek [27] found that the susceptibility of *L. dispar dispar* to its native baculovirus (Lymantria dispar multiple nucleopolyhedrovirus, or LdMNPV) increased with increasing larval density, in a range from 1 larva to 20 larvae per rearing cup. However, this study was carried out with larvae that were fed a diet that had been inoculated with the virus. A more likely scenario in our experiments would be the reactivation of latent baculovirus infections to an acute phase caused by the stress of rearing larvae at higher larval densities, a phenomenon that has been reported previously [28,29]. An examination of reactivation of latent LdMNPV infections in *L. dispar asiatica* larvae by Pavlushin et al. [18] did not detect any impact of larval density on LdMNPV-mediated mortality or larval hemolymph phenoloxidase levels. The LdMNPV-mediated mortality in the Pavlushin et al. study amounted to 4% of both the solitary and crowded test populations. We did not observe cadavers in our study exhibiting signs of nuclear polyhedrosis, although we cannot exclude the possibility that there were LdMNPV-killed larvae at densities of seven and nine larvae/cup that we did not detect. While the Pavlushin et al. study used specimens from field-collected egg masses, our specimens were derived from established laboratory colonies in which baculovirus-mediated mortality has not been observed.

The Pavlushin et al. study identified a four-fold increase in dopamine, an insect stress hormone [30], in *L. dispar asiatica* larvae reared alone compared to larvae reared in a group of 10/container. This result points to the possibility of additional physiological factors that may account for observations of suboptimal survival, pupation and emergence that we observed at both low densities (e.g., the Allee effect) and high densities.

Data for the three populations followed the same trends, suggesting that responses to changes in larval density are not radically different among different subspecies, biotypes, or populations of *L. dispar*. Although the trends were the same, differences were observed among the three populations at any given density. These differences may reflect the relative success with which different populations have adapted to the artificial diet used in these experiments. The temperature used for rearing may have also influenced the performance of larvae from the different populations. The average annual minimum and maximum temperatures of Guizhou (6.8 ± 0.12 and 18.1 ± 0.14 °C from 2005 to 2015; http://data.cma.cn/) and Connecticut (4.2 ± 0.33 and 15.8 ± 0.39 °C; https://www.ncdc.noaa.gov/) are more similar to each than to the average annual minimum and maximum temperatures of Hebei (−0.2 ± 0.27 and 23.2 ± 0.41 °C; http://data.cma.cn/). The Hebei larvae exhibited the highest survival and emergence rates at the temperature used for rearing (25 ± 0.5 °C).

## 5. Conclusions

This study examined the response to increasing larval density in three different colonies of *L. dispar,* representing two different subspecies in different biotypes. Effects on the duration of larval development, larval survival rate, pupation rate, and emergence rate all followed the same two-tailed trend in all three colonies. These experiments were carried out in a laboratory setting and thus controlled for ecological factors that affect developmental traits, such as environment, climate, and natural enemies. As such, this study raises questions about other genetic or physiological factors that control density-dependent effects on gypsy moth development. In addition, the results of this study have implications for the rearing of gypsy moth in culture.

## Figures and Tables

**Table 1:** Effect of larval density on the development and survival of gypsy moth Lymantria dispar evaluated by a two-way ANOVA for the total larval duration, larval survival rate, pupation rate, and emergence rate.

Parameters	Factor	Two-Way ANOVA		
		df	F ( DFn, DFd )	*p*-Value
Larval duration (d)	Larval density (*n*/cup)	4	F (4, 30) = 20.73	<0.0001
	Population	2	F (2, 30) = 14.99	<0.0001
	Larval density (*n*/cup) × population	8	F (8, 30) = 0.79	=0.6177
Larval survival rate (%)	Larval density (*n*/cup)	4	F (4, 30) = 109.1	<0.0001
	Population	2	F (2, 30) = 53.62	<0.0001
	Larval density (*n*/cup) × population	8	F (8, 30) = 1.123	=0.3767
Pupation rate (%)	Larval density (*n*/cup)	4	F (4, 30) = 68.50	<0.0001
	Population	2	F (2, 30) = 50.39	<0.0001
	Larval density (*n*/cup) × population	8	F (8, 30) = 0.89	=0.5313
Emergence rate (%)	Larval density (*n*/cup)	4	F (4, 30) = 106.2	<0.0001
	Population	2	F (2, 30) = 9.507	<0.0001
	Larval density (*n*/cup) × population	8	F (8, 30) = 0.52	=0.8332

## Data Availability

The data presented in this study are available on request from the corresponding author.
